# Health and Social Care Professionals' Perspectives of the Desired Interprofessional Collaboration in Frequent Attenders' Person‐Centred Care: A Qualitative Study

**DOI:** 10.1111/scs.70217

**Published:** 2026-03-10

**Authors:** Kaisa Hartikainen, Minna Elomaa‐Krapu, Leena Salminen, Miia Laasanen, Heli Virtanen

**Affiliations:** ^1^ Department of Nursing Science University of Turku Turku Finland; ^2^ Metropolia University of Applied Sciences Helsinki Finland; ^3^ Futureproof Health and Wellbeing Innovation Hub Metropolia University of Applied Sciences Helsinki Finland; ^4^ Turku University Hospital Turku Finland; ^5^ Faculty of Medicine University of Turku, Sote Academy Turku Finland

**Keywords:** a qualitative study, frequent attenders, integrated care, interprofessional collaboration, person‐centred care

## Abstract

**Aim:**

The aim of the study was to describe the desired nature of interprofessional collaboration (IPC) in person‐centred care (PCC) for frequent attenders (FA). The study employed a futures thinking approach to create an alternative vision of the future, aiming to understand how professionals should collaborate across organisational boundaries to achieve successful integration in person‐centred care for FAs.

**Methods:**

The semi‐structured interviews with social and healthcare professionals (*n* = 38) delivering care and services for frequent attenders were conducted in three public health and social care organisations in Southern Finland. Inductive content analysis was used. Ethical approval was obtained from the Ethics Committee for Human Sciences at the University of Turku, and research permissions were secured from all participating organisations. Research participants confirmed their voluntary participation through electronic informed consent.

**Results:**

Social and health care professionals described the desired interprofessional collaboration in person‐centred care for FAs through a unified operating culture and values and integrated and adaptive care management. It was described through continuous co‐creation of joint networks, outreach interprofessional practices and fostering a collaborative culture among social and healthcare professionals and other stakeholders across organisations. FAs and their close ones are seen as key members of the interprofessional team, collaborating with professionals to construct and maintain a holistic view of the FA, ensuring seamless service interfaces.

**Conclusion:**

In the future the desired IPC can be achieved by co‐creating and implementing cross‐organisational practices and investing in nurturing innovation‐oriented professionals who serve as bridge‐builders across sectors. Furthermore, interprofessional learning, the involvement of experts by experience, and training in co‐creation and flexible networking skills enhance professionals' capacity to address the complex needs of FAs in the future.

## 1 Introduction

One main challenge for healthcare systems in Europe is the care management of individuals with multimorbidity [1], which may lead to extensive use of services, and frequent attendance [2]. These persons, frequent attenders (hereafter FAs) [3], contribute a growing burden on the healthcare system [4, 5] by generating a large amount of the costs of social and healthcare services [5, 6, 7] and are responsible for the increased workload in healthcare [8]. In Europe, FAs account for 30%–50% of all consultations with healthcare providers [8] requiring a wide range of social and health services [9, 10, 11]. In this context, FAs challenge healthcare systems as regards delivering high‐quality, cost‐effective health services that cater to individuals' unique needs and enhance their overall service experience. This study identifies the integration of services as a solution for achieving person‐centred care (hereafter PCC) for FAs.

The World Health Organisation (WHO) supports global efforts to advance integrated and person‐centred healthcare services [12]. For the care of FAs, the requirements for integration extend beyond healthcare to include intersectoral collaboration with social services and other stakeholders [12, 13]. In Finland, the reform of the social and healthcare system aims to improve coordinated and individualised services, particularly for FAs [9]. Under this model, eighteen well‐being services counties are responsible for organising care, defining service packages and pathways tailored to various client needs. A service pathway of FA may involve multiple providers across public, private and third sector organisations [14].

While systemic integration of social and health services addresses service fragmentation, successful PCC also requires integration at the meso (organisational/professional) and micro (clinical) levels [13]. For FAs, this entails cross‐organisational service integration and the involvement of multiple professionals throughout the care process [9, 15]. Achieving this level of integration necessitates interprofessional collaboration (hereafter IPC) and shared responsibility among professionals within and across organisational boundaries to tailor integrated care processes and ensure continuous, coordinated care delivery [10, 13]. In this study, IPC is defined as the collective engagement of various health and social care professions in responding to the needs of FAs and providing integrated services. It is characterised as a dynamic and interactive process grounded in interdependence, shared authority and partnership among all involved professionals [16].

Despite the paradigmatic shift towards service integration, person‐centredness is poorly implemented in the care of FAs, and the provided care appears to address their needs inadequately. FAs still encounter difficulties in resolving their situations and often find themselves caught in service loops [17]. The challenges are associated, for example, with FAs' experiences of feeling undervalued, receiving care that does not meet their expectations, and being repeatedly referred back and forth between healthcare professionals without a solution [18]. The perceived challenges are also related to professionals' experiences in providing appropriate care for FAs, which stem from issues such as complicated care coordination, lack of communication and information sharing with other healthcare professionals, and the multifaceted care needs of FAs that are unmanageable in unprofessional visits [19]. Although healthcare systems worldwide have reoriented their mission to implement care integration to enhance person‐centredness, the realisation of service integration that effectively addresses the personal needs of FAs remains a significant challenge.

Delivering PCC depends on the ability of professionals to meet individual needs, ensuring tailored services and a seamless continuum of care, particularly in populations with a growing disease burden, where professionals from various disciplines and sectors must collaborate to integrate services [20]. Despite the numerous programmes and initiatives strengthening integration at the professional (meso) and clinical (micro) levels [12, 21, 22] and promoting interprofessional education [23] and interprofessional competence of professionals [24, 25], the implementation of IPC remains challenging in various healthcare settings [26, 27, 28]. The facilitators and barriers to IPC are also well known, and multiple tools and guidelines for its successful implementation have been developed [20, 26]. While the research has explored the experiences and perspectives of professionals regarding IPC in various healthcare settings, it mainly focuses on current practices and perceptions of existing IPC [27, 29, 30, 31, 32]. The features of desired IPC in PCC for FAs are poorly defined, and the perspectives of health and social care professionals in primary and specialised care remain largely unknown. Instead of focusing on current practices and experiences, there is a need for future‐oriented research that looks further ahead and creates a visionary picture of the IPC required to achieve person‐centred and integrated care.

This study uses futures thinking to create an alternative vision of the future, moving beyond historical continuities [33]. Futures thinking is an anticipatory and reflective approach that allows alternative and desired futures to be explored to inform strategy, foster innovation and guide transformative change [34]. Whereas traditional methods view the future as a continuation of current trends, futures thinking recognises it as open, uncertain and subject to collective agency, thus encouraging research questions that challenge prevailing assumptions and actively explore alternative futures. Previous research suggests that strengthening IPC relies on joint discussions and co‐creation among professionals, leading to a more comprehensive understanding of IPC in PCC for FAs [27, 30]. In this study, a futures thinking approach is employed as a methodological framework to guide both data collection and analysis. Rather than merely describing existing practices, this approach facilitates the co‐creation of alternative and desirable future scenarios for IPC in PCC for FAs. It also informs the analysis by systematically identifying those envisioned by the participants. By collaboratively defining the desired IPC, it is possible to identify how existing practices at both micro and meso levels need to be developed to achieve successful integration. This study aimed to describe the desired nature of IPC in the PCC for FAs.

## 2 Methods

### 2.1 Research Design

The research was conducted as a triangulation of individual [35], dyadic [36] and focus group interviews [37] with social and healthcare professionals (*n* = 38) delivering care and services for FAs. The study was conducted following the Consolidated Criteria for Reporting Qualitative Research (COREQ) checklist [38].

### 2.2 Participants and Recruitment

A purposeful sampling strategy [39] was used to recruit health and social care professionals from three public health and social care organisations in Southern Finland. These organisations employ diverse professionals from social and healthcare sectors, offering varied perspectives across services. As part of a broader healthcare reform, they integrate services to improve efficiency and transform practices within and between primary care and other health and social service organisations [40, 41].

The researcher initiated recruitment: first, by contacting a contact person in one of the organisations and disseminating information via the intranet; second, by presenting the study to managers or personnel in two of the organisations. In all three organisations, contact persons distributed the electronic invitation letter, the participant information sheet and the privacy statement to employees belonging to the target group of the study. The inclusion criteria for interviewees were: (i) a social or healthcare profession and (ii) regular work with frequent attenders. The researcher verified eligibility before conducting interviews. No participants withdrew from the study.

The study participants (Table 1) encompassed various professionals, including registered nurses, physicians, psychotherapists, social workers, psychologist, rehabilitation counsellor, physiotherapists, public health nurses, practical nurses and occupational therapists. They primarily worked with working‐age and older adults frequently using social and healthcare services.

**TABLE 1:** Interview formats and participants.

Interview format	Participants
Individual interviews (*n* = 4)	Chief physician (*n* = 1)
	Social worker (*n* = 1)
	Physiotherapist (*n* = 1)
	Registered nurse (*n* = 1)
Dyadic interviews (*n* = 5)	Physiotherapists (*n* = 3)
	Registered nurses (*n* = 2)
	Physician (*n* = 1)
	Psychotherapist (*n* = 1)
	Rehabilitation counsellor (*n* = 1)
	Dentist (*n* = 1)
	Practical nurse (*n* = 1)
Focus group interviews (*n* = 5)	Social workers (*n* = 7)
	Registered nurses (*n* = 4)
	Occupational therapists (*n* = 4)
	Public health nurses (*n* = 2)
	Practical nurses (*n* = 2)
	Physiotherapists (*n* = 3) psychologist (*n* = 1)
	Rehabilitation counsellor (*n* = 1)
14 interviews	38 professionals

### 2.3 Data Collection

Data was collected through semi‐structured interviews [42] between December 2022 and April 2023, including 14 interviews: five dyadic, five focus groups and four individual interviews (Table 1). Interviews continued until data saturation was achieved, with no new themes emerging [43].

Various interview formats were offered to facilitate participants' involvement in the study. Dyadic interviews and focus groups were used to encourage interaction and co‐create collective understanding about the desired IPC for implementing PCC for FAs [36, 37]. Focus groups ranged from three to eight participants. Due to scheduling conflicts, four interviews were conducted individually. A flexible approach was used to respect participant expertise, ensure equal access and promote rich data collection [44].

A semi‐structured interview format was used to maintain a consistent thematic approach across interviews [42]. The themes, based on the key elements of PCC [45], explored perspectives of professionals on the desired IPC: (1) individualised and holistic focus, (2) care coordination, (3) shared decision‐making and (4) communication. Futures thinking was implemented in data collection through visioning [46], guiding participants to co‐create the desired future of IPC, drawing on current practices only to inform this vision. If participants began to focus on current practices, the interviewer gently redirected the conversation towards desired futures by posing follow‐up questions such as, ‘If there were no limitations, what could this look like in the future?’ The Protocol Refinement (IPR) Framework guided the design and conduct of the interviews [47]. A preliminary interview was conducted to assess the structure, clarity and duration; the data were included in the final dataset as no significant changes were needed.

The researcher assigned the pairs and focus groups based on the organisation's internal structures, accommodating participants' preferences to be interviewed with their work partner or team. Nine of the ten pairs or focus groups included diverse professionals. The interviews were conducted face‐to‐face within organisations or via secure videoconferencing (Zoom), with the method chosen collaboratively with the participants. One researcher (KH) conducted all interviews, which lasted 56 to 72 min (total 872 min) and were audio‐recorded.

### 2.4 Qualitative Data Analysis

All data, including individual, dyadic and focus group interviews, were analysed together using inductive content analysis [48], without differentiating between interview formats. After the interviews, recordings were transcribed verbatim (269 pages of transcribed material, font: Calibri 11, single‐spaced) and the data were anonymised.

**FIGURE 1:**
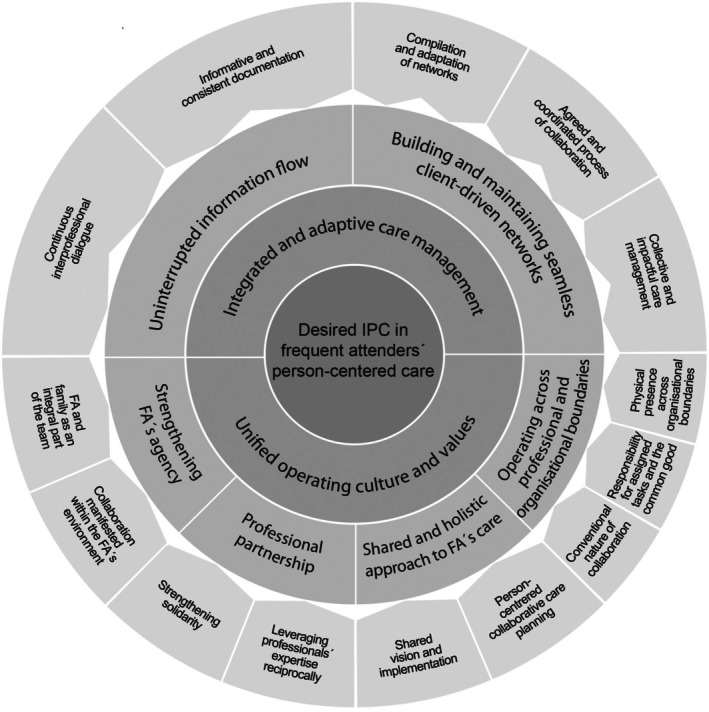
Desired IPC in Frequent Attenders' Person‐Centred Care.

The data analysis followed an inductive and iterative approach, with repeated reviews to refine the precision of the analysis with each iteration. Initially, the data was listened to and read multiple times to explore participants' perspectives on their desired IPC in the care for FA. Only expressions explicitly relating to desired IPC were extracted and subjected to analysis. Original expressions (*n* = 382) were documented on a Word table, condensed and coded while preserving essential content (K.H.). Similarities and differences were analysed to form descriptive sub‐categories (K.H.). After this stage, two researchers (K.H. and M.E.‐K.) independently reviewed the analysis, engaging in discussion and subsequently modifying the names of the generated sub‐categories. The iterative process continued by merging sub‐categories into categories and main categories to increase abstraction (K.H.). The formation and naming of categories were critically examined through reflective discussions among two researchers (K.H. and M.E.‐K.). In the final stage of the analysis, the entire research team independently examined the data and subsequently refined through collective discussion, negotiation and consensus‐building. To ensure qualitative trustworthiness [49, 50], regular team meetings were held to reflect on data interpretation, with consensus reached through discussion. The professional backgrounds of all team members in nursing, rehabilitation and education, as well as their relationships to the topic, were recognised and considered throughout the research process. The main categories, categories and sub‐categories are shown in Figure 1.

### 2.5 Ethical Considerations

Ethical approval (29/2022) was obtained from the Ethics Committee for Human Sciences at the University of Turku, and research permissions were secured from all participating organisations. Research participants were informed about the study both in written and oral forms, with voluntary participation confirmed through electronic informed consent. Participants' rights and data processing were respected throughout the study [51]. The focus group interviews were conducted in a private setting exclusively among the participants [52]. No dependencies existed between participants and the researcher.

## 3 Results

In delineating their perspectives, professionals described the desired IPC in PCC for FAs (Figure 1) through the following two main categories: Unified operating culture and values and Integrated and adaptive care management.

### 3.1 Unified Operating Culture and Values

The main category of Unified operating culture and values was described as Strengthening the FA's agency, Professional partnership, Shared and holistic approach to FA's care and Operating across professional and organisational boundaries.

#### 3.1.1 Strengthening FA's Agency

Social and health care professionals described that they desired an IPC with a broader networked activity to strengthen the FA's agency. This meant including the FA and the family as integral parts of the team and manifesting collaboration within the FA's environment.

The FA's and family's integration into the team was envisioned as equal agency alongside professionals within an interprofessional team. Their presence and involvement should be ensured in interprofessional meetings where the FA's situation is discussed. Strengthening the FA's agency involves collaboration in their environment, where professionals from different organisations or units jointly meet the FA, assess their needs, plan care and ensure care continuity together with the FA and other professionals.

We could do home visits together and look at the home and carry out the assessment together with the physiotherapist. And the home care workers would also share their thoughts (about the FA's situation). I9

#### 3.1.2 Professional Partnership

According to the professionals, the desired IPC was envisioned as a professional partnership, which consists of strengthening solidarity and leveraging professionals' expertise reciprocally.

Strengthening solidarity was envisioned as fostering a culture of IPC, breaking down silos between professionals and organisations and promoting equality and respect for diverse expertise. The desired IPC was demonstrated through ongoing discussions on concrete collaboration, identifying common interfaces and moving beyond discipline‐specific approaches. Ideally, IPC operates with a low hierarchy, where professionals collaborate as partners committed to the best interests of FAs and share responsibility for challenging decisions.

We would understand each other's professional skills. That we are all at the same level, even though one might have more education or more work experience, but everyone is doing the same job. We have that one person there who needs to be rehabilitated one way or another. (I8)

Leveraging professionals' expertise reciprocally was envisioned in three ways. First, it involved utilising all professionals' expertise within a dialogical atmosphere, where diverse perspectives are valued, and belittling of expertise is avoided. Second, it was envisioned as the professionals' ability to identify each other's skills and appropriately direct FAs to the right professional, ensuring shared responsibility and continuity of care across transitions. Third, the leveraging of professionals' expertise was envisioned as the mutual exchange of advice, feedback and assistance. This included positive reinforcement, constructive feedback for professional growth, and interprofessional consultations when facing uncertainty about how to proceed with a FA.

#### 3.1.3 Shared and Holistic Approach to FA's Care

The desired IPC was envisioned as a shared and holistic approach to FA's care, which includes a shared vision and implementation, as well as person‐centred collaborative care planning.

A shared vision and implementation were envisioned as a mutual understanding of collaboration, including its significance, goals and means of achievement. Ideally, professionals should continuously strive for a unified approach to FA's care, align on common objectives, and direct their efforts accordingly. Most desirably, they should adopt a shared framework they rely on and commit to, fostering consistent practices and approaches to FA's care. The desired IPC should also be characterised by mutual, implicit trust, with the fundamental belief that all professionals are committed to the shared goal of supporting FA.

Person‐centred collaborative care planning was envisioned as a shared commitment to comprehensively assess the FA's situation, conduct shared evaluations, plan care and make decisions based on a common, holistic understanding of the person. Decision‐making should involve the FA, professionals and administrative decision‐makers, leveraging the expertise of all participants. It is particularly essential to utilise the perspectives of professionals who have worked with the FA for a longer period and can evaluate the FA's situation over time. Ideally, a holistic approach would bring together various professionals and the FA, enabling collaborative problem‐solving that goes beyond professional and organisational boundaries. If the FA's situation changes, it should trigger a collective response and a new cycle of planning and decision‐making.

We would see the FA as shared, and not in a way that the FA's foot is my thing and the nose can be your thing, but rather that the FA is whole and entirely shared by us, and we would solve it (the FA's situation) together with the FA. I6

#### 3.1.4 Operating Across Professional and Organisational Boundaries

In operations that merge across professional and organisational boundaries, the desired IPC was envisioned in descriptions of the conventional nature of collaboration, responsibility for assigned tasks and the common good, and professionals' physical presence across organisational boundaries.

The desired nature of conventional collaboration was envisioned as the routine daily activities of professionals and genuine teamwork between professionals and FAs. Participants envisioned IPC as a natural, regular and automatic interprofessional activity in various situations, often without the need for specific agreements. Ideally, both professionals and FAs would experience care as a unified effort by an interprofessional team, rather than isolated actions by individual professionals.

It (IPC) is not something special, somewhat strange and peculiar, or very rarely occurring, nor does it require special arrangements. I14

Responsibility for assigned tasks and the common good involves professionals' accountability for their designated care tasks and their flexibility in taking on tasks beyond their expertise. In the desired IPC, this could manifest as proactive engagement and the avoidance of unnecessary shifting of responsibilities between professionals or organisations. Ideally, professionals are prepared to take on tasks traditionally assigned to other roles, as well as duties that fall within the general competence of any team member.

Professionals envisioned physical presence across organisational boundaries as involving various stakeholders and professionals working outside their own organisations. Ideally, this would include professionals moving between organisations to attend interprofessional care meetings. Additionally, this presence was expected to enable low‐threshold interactions for exchanging updates and offering expertise to other stakeholders. The physical presence of professionals was described as strengthening the formation of interprofessional networks in FA's care.

### 3.2 Integrated and Adaptive Care Management

The main category of ‘Integrated and Adaptive Care Management’ was described as Building and maintaining seamless client‐driven networks and Uninterrupted information flow.

#### 3.2.1 Building and Maintaining Seamless Client‐Driven Networks

The desired IPC was envisioned as a seamless, client‐driven network building and maintenance which includes the compilation and adaptation of networks, an agreed and coordinated process of collaboration, as well as collective and impactful care management.

Professionals envisioned the compilation and adaptation of networks as a client‐driven process, involving the construction of networks composed of various stakeholders and their reactive adaptation to the evolving needs and circumstances of the FA. This approach ensures that IPC will be individually tailored to each FA, irrespective of internal organisational processes or inter‐organisational procedures.

The agreed and coordinated collaboration process was envisioned as ongoing mutual agreements among professionals across organisations, and maintaining a shared, comprehensive 360‐degree overview. This should include agreements regarding the initiation, implementation and conclusion of IPC. Furthermore, professionals should collectively form an up‐to‐date overview of all stakeholders involved in the FA's care. By making mutual agreements on roles, tasks and schedules, the process would ensure timely participation of each professional and prevent overlapping in interventions, promoting efficient and coordinated care.

Collective and impactful care plan management was envisioned as a collaborative effort where professionals share responsibility for the FA's care and implementation of IPC during the process. Shared responsibility should involve active management, advancement and monitoring of the FA's care according to an agreed schedule, including reporting on the implementation and effects of care within an interprofessional network. The desired IPC was described as seamless, with the FA's matters transitioning from one professional to another on a ‘hand‐to‐hand’ basis. Additionally, professionals should share joint responsibility for building and continuously developing collaboration, enhancing teamwork within interprofessional teams and across the FA's service pathway.

#### 3.2.2 Uninterrupted Information Flow

The desired IPC involved uninterrupted information flow, built on continuous interprofessional dialogue and informative and consistent documentation.

Professionals envisioned continuous interprofessional dialogue in two ways. First, it was visualised as open, low‐threshold discussions, which could include formal reporting, the exchange of informal thoughts, and the validation of shared understanding of agreed matters regarding the FA's care. The key aspects should be the regularity of dialogue and professionals' genuine willingness to share knowledge.

The interprofessional collaboration would require a conversational culture. Not just in a way that a certain matter is merely provided as information to another professional, but rather that it includes dialogue. I9

Second, the desired interprofessional dialogue was envisioned as continuous information flow regarding care progression and any changes within the network of professionals, the FA, and their family. This dialogue is especially important when the FA's care deviates from the joint plan or when changes in the FA's condition affect their care or the actions of other professionals involved.

Professionals envisioned informative and consistent documentation as clear and up‐to‐date patient records using jointly agreed structures and terminology. In the future, documentation should be comprehensible, avoiding profession‐specific jargon, with real‐time, collective access to all relevant information. Co‐creating patient records that incorporate the FA's perspectives should be implemented. These approaches support consistent operational models, fostering commitment to the FA's care goals across professional and organisational boundaries.

## 4 Discussion

The study aimed to describe the desired nature of IPC in the PCC for FAs utilising a futures thinking approach. In contrast to earlier research primarily describing existing practices of IPC [27, 29, 30, 31, 32], this study employs a novel, forward‐looking and co‐creative approach that enables professionals to collaboratively envision desired futures, thereby offering new methodological perspectives for IPC in the PCC for FAs. The results show that the desired IPC is envisioned as a unified operating culture and values and integrated and adaptive care management. A unified operating culture and values emphasise strengthening the FA's agency, fostering mutual partnerships among professionals, adopting a shared and holistic approach to FAs' care, and conducting interprofessional operations across organisational boundaries. Furthermore, integrated and adaptive care management involves creating and maintaining seamless client‐driven networks and ensuring uninterrupted information flow among all stakeholders.

This study highlighted the importance of the participation of the FA and their close ones as an essential part of an interprofessional team. The IPC was not only about working together among themselves but also about including FAs and their close ones as integral team members. Moreover, the importance of implementing IPC within the FAs' environment was emphasised. The foundation of person‐centredness was highlighted by acknowledging the participation of FAs and their active role as experts in their own care [45, 53]. However, there still appears to be a gap in the desired behaviours and actions of professionals through which the involvement of FAs is effectively realised. Despite the study confirming a paradigmatic shift in the value base of professionals towards IPC between professionals and FAs, previous studies show that the experiences of FAs regarding involvement and expertise in their care remain poor [17, 18]. FAs continue to feel unheard and undervalued and perceive a lack of respect from professionals [54]. Sharabani et al. [55] found that FAs still often perceive healthcare personnel as professionals with authority and knowledge, similar to parents, while also seeing them as supportive individuals capable of guiding and managing their care. This may be because changing the role of FAs from a passive recipient of care to an active participant requires expertise from both the perspectives of professionals and FAs to engage in IPC in a new way. In the future, it will be important to further develop professionals' competencies in strengthening FAs' agency. This is achieved, for example, by increasing the involvement of experts by experience and implementing more interprofessional simulations both during degree programmes and as part of continuing education for professionals.

This study challenges organisations and professionals to reassess the implementation strategies for IPC. The nature of professionals' work must become increasingly mobile to replace traditional practices, where interprofessional meetings are typically structured around organisational priorities and professional terms. The key element to enhance IPC and care integration seems to be the development of outreach interprofessional practices and team functions beyond organisational boundaries. This is a complex phenomenon that requires actions on multiple levels, including addressing cultural and economic issues [56], ensuring adequate support in terms of time and space for collaboration [57] and making significant efforts to promote open and active communication beyond organisational boundaries [58]. Such communication is essential for co‐creating shared goals [58], clarifying professionals' roles and strengthening professionals' experience of working together as a team [28], even when they are not part of the same organisation. Karam et al. [28] identified informal communication and developing interpersonal relationships as critical factors in fostering team formation among professionals. These processes are significantly supported by physical proximity and by sharing a common organisational culture that could more easily occur when professionals work within the same organisation [28]. These aspects are particularly challenging in FAs' care, where interprofessional teams are formed across organisations [9]. To achieve meaningful change in IPC in the PCC for FAs, policy‐level guidance and appropriate incentives such as targeted funding may be needed to require and support organisations to jointly develop outreach interprofessional practices alongside their internal processes. In order to overcome the challenges associated with the care for FAs, organisations should appoint coordinators who possess their expertise and authority to identify key stakeholders in the collaborative network from the perspective of FAs and to organise and lead the co‐development of cross‐organisational practices together with professionals from different organisations, FAs and their families, and organisational leadership, utilising futures‐oriented and co‐creative approaches. At the practical level, within IPC in the PCC for FAs, these coordinators could facilitate communication and team building across various levels, organise meetings and ensure the smooth exchange of information [58, 59].

This study indicated that the desired IPC in FA's care is characterised by interprofessional partnerships, which are envisioned as having mutual equality, reciprocal respect and a continuous strengthening of solidarity among social and healthcare professionals. The desired IPC involves professionals independently seeking and building interfaces and networks. Due to the highly individualised needs of FAs, their care networks and teams are extensive, constantly evolving and require professionals to continuously reshape them collaboratively. Reflecting on this issue, it seems that the structural collaboration processes between organisations and units lack the necessary multidimensionality and flexibility for PCC for FAs. Furthermore, solidarity and partnership in FA's care do not naturally emerge from existing structures and processes but require professionals' active efforts to build a shared value base, form teams and foster collaboration to integrate FA's care across organisational boundaries. The success of IPC depends on the ability of professionals to work collaboratively, and core competencies needed in IPC have been defined in various settings [25, 60, 61, 62]. These competency frameworks emphasise domains such as role clarification, teamwork, communication, values and ethics, shared decision‐making [25, 60, 61, 62], client orientation [62, 63] and ensuring a seamless service path [63]. However, relatively little attention has been given to competencies related to building and developing interprofessional networks and teams or enhancing the co‐creation of a shared value base and team spirit. It appears that, in the future, it will be critically important to strengthen the competencies of all social and healthcare professionals in facilitating relationships [61], fostering their transformative competencies [64], and transversal skills [65] to reshape and transform IPC to meet the needs of FAs. Unfortunately, at the curricular level, a substantial portion of education related to IPC is still delivered within the silos of individual degree programmes or even as self‐directed online courses [66]. Consequently, considerable effort will be required to move away from uniprofessional curricula in higher education [67] and to implement genuine interprofessional training at both undergraduate and postgraduate levels [31, 67].

The study highlighted the importance of the following aspects: interprofessional low‐threshold dialogue, smooth information flow, fluent negotiation about collaboration and its implementation, and collaborative care planning in the care of FAs. Professionals emphasised that a key aspect of IPC in FA's care should be a real‐time, comprehensive 360‐degree view of the FA and their care, built and sustained through ongoing discussions and shared knowledge formation between professionals and the FA. It appears that professionals' description of the desired IPC reflects a low‐threshold and practice‐oriented co‐creation approach. Joint co‐creation processes [68] beyond organisations should be built on professional‐led collaborative actions [69] and bottom‐up strategies [31]. Low‐threshold IPC is a complex combination of individual and environmental factors [59]. Timperi et al. [59] found that individual commitment and professionals' active involvement in collaboration can enhance the organisation's overall appreciation of cooperation and support the development of inter‐organisational collaboration. Conversely, the values and culture of organisations may hinder the formation of a collaborative climate and negatively influence professionals' attitudes and personal motivation to collaborate beyond organisational boundaries [59].

Some recommendations for clinical practice and education can be made based on the results. By increasing interprofessional learning and the involvement of experts by experience during undergraduate education, future professionals' ability to promote the agency of FAs and their skills for collaboration across organisational boundaries can be strengthened. Training that focuses on developing competencies in co‐creation, building interprofessional networks and working flexibly within these networks supports future professionals' capacity to meet the complex and evolving needs of FAs. Moreover, organisations must foster an environment that promotes collaboration across organisational boundaries and lays the foundation for the co‐creation of interprofessional team cohesion. Accordingly, organisational leaders should actively promote IPC by allocating resources for joint initiatives across organisational boundaries. Providing interprofessional continuing education across organisations is an appropriate means to strengthen collaborative skills and foster successful IPC in clinical practice. Finally, organisations need to empower collaboration‐oriented professionals with the authority to adapt their work and develop systemic, client‐driven and effective networks beyond organisational and unit‐level processes.

### 4.1 Strengths and Limitations

A key strength of this study is the diverse representation of participants from various professional groups who were experienced by the phenomenon under study [70] with a substantial sample size (*n* = 38). Moreover, two researchers (KH and ME‐K) were involved in the analysis to facilitate dialogue and negotiation throughout the process. The consensus on the analysis was co‐created by the research team to strengthen its credibility [48].

The study has limitations in participant selection. It focused on public sector professionals, excluding those from the private and third sectors, which limits the diversity of perspectives and the transferability of the findings to other contexts [49]. Including professionals from these sectors could have enriched the data [48]. However, participants represented both social and healthcare sectors, covering various organisations and primary and specialised care and described the desired IPC extending beyond organisational boundaries, including collaboration with private and third‐sector professionals. Additionally, selection bias may have occurred, as the study might have attracted professionals who were already highly engaged in collaborative practices.

The study utilised various interview formats, which could introduce bias due to differing information yield. Individual and dyadic interviews provided in‐depth insights, while focus groups promoted interprofessional discussions. However, focus groups posed a risk of dominant voices influencing the conversation [44], which was mitigated by ensuring equal participation. In addition, despite its strengths in fostering creativity and forward‐looking perspectives, the futures thinking approach is limited by its abstractness and participants' varying readiness for future‐oriented thinking [34]. As a result, some participants may have tended to describe existing IPC practices instead of imagining fundamentally desired futures.

## 5 Conclusion

Achieving the desired IPC requires social and healthcare organisations to systematically develop and implement cross‐organisational practices and foster professionals who build collaborative networks across sectors. Integrating interprofessional learning, the involvement of experts by experience, and training in co‐creation and flexible networking skills into social and healthcare education enhances professionals' ability to meet the evolving needs of FAs. The study highlights the potential of the futures thinking approach and co‐creation for advancing IPC in PCC for FAs. In addition, the futures thinking approach also serves as a qualitative data collection method. Further research is needed in more diverse contexts, with particular emphasis on the development and implementation of desired IPC in the care for FAs across organisations.

## Author Contributions

The study was designed by Kaisa Hartikainen, Minna Elomaa‐Krapu, Leena Salminen and Heli Virtanen. Kaisa Hartikainen performed data collection. Data analysis was conducted by Kaisa Hartikainen with contributions from Minna Elomaa‐Krapu and with analytical input from Leena Salminen and Heli Virtanen. Kaisa Hartikainen drafted the manuscript, which all authors critically revised and approved.

## Funding

This work was supported by Finnish Diabetes Association (https://doi.org/10.13039/501100008415), The Finnish Nursing Education Foundation sr (https://doi.org/10.13039/501100020271) and The Okka Foundation.

## Ethics Statement

Ethical approval (29/2022) for the study was obtained from the Ethics Committee for Human Sciences at the University of Turku and research permissions were sought from all participating organisations before the initiation of the study. Research participants were informed about the study in written and oral forms and participation was voluntary. All participants provided written informed consent before participating in the study. Participant's rights and the processing of their data were respected during the study.

## Conflicts of Interest

The authors declare no conflicts of interest.

## Data Availability

Research data are not shared due to privacy or ethical restrictions.
